# RGD peptide promotes follicle growth through integrins αvβ3/αvβ5 in three-dimensional culture

**DOI:** 10.1530/REP-24-0151

**Published:** 2025-01-02

**Authors:** Cassandra Matsushige, Kaelyn Kitazumi, Amanda Beaman, Marissa Miyagi, Michelle D Tallquist, Yukiko Yamazaki

**Affiliations:** ^1^ Yanagimachi Institute for Biogenesis Research, Department of Anatomy, Biochemistry and Physiology, John A Burns School of Medicine, University of Hawaii at Manoa, Honolulu, Hawaii, USA; ^2^ Center for Cardiovascular Research, Department of Medicine, John A Burns School of Medicine, University of Hawaii at Manoa, Honolulu, Hawaii, USA

**Keywords:** RGD peptide, integrins ⍺vβ3/⍺vβ5, *in vitro* follicle growth, three-dimensional culture

## Abstract

**In brief:**

Three-dimensional ovarian tissue culture is a unique model to define the effects of molecules on folliculogenesis. Using this model, we determined that RGD–integrin interaction plays a role in antrum formation and theca cell differentiation.

**Abstract:**

We recently developed a three-dimensional (3D) ovarian tissue culture system supported by bacterial-derived dextran hydrogel. Arg-Gly-Asp (RGD) is an extracellular matrix-derived triple peptide. Immature ovarian tissues cultured in RGD-modified dextran hydrogel significantly promoted antral follicle growth and oocyte quality compared with those cultured in dextran hydrogel alone. In this study, we examined the mechanism of follicle growth stimulated by RGD treatment in the 3D system. First, we detected that direct contact between RGD-modified dextran hydrogel and ovarian interstitial cells is necessary to promote antral follicle growth. Therefore, we hypothesized that RGD stimulates antral follicle growth through RGD-binding integrin receptors expressed in the interstitial cell mass. Using quantitative PCR (qPCR) and immunochemical staining, we identified that integrins ⍺vβ3 and ⍺v5 are predominantly expressed in the ovarian interstitial compartment. To assess the effect of RGD–integrin interaction on follicle growth, ovarian tissues were cultured with cilengitide (Ci), an inhibitor specific for ⍺vβ3 and ⍺vβ5. Ci treatment suppressed RGD-induced follicle growth and oocyte quality in a dose-dependent manner. When the interstitial cell aggregates were cultured with RGD, cell migration and theca-related gene expression were significantly upregulated. Ci treatment dramatically suppressed these RGD-induced activities. In coculturing the interstitial aggregate and secondary follicles with RGD, migrating cells formed the outermost cell layers around the follicles, like theca layers, which were totally blocked by Ci treatment. In conclusion, our results suggest that RGD stimulates theca cell differentiation in the ovarian interstitial cells through integrins ⍺vβ3 and ⍺v5 to promote antral follicle growth in our 3D system.

## Introduction

In the mammalian ovary, the follicle is the fundamental unit. It is composed of three different types of cells: the oocyte, granulosa and theca. The follicle plays a major role in the dual function of the ovary, namely, oocyte maturation/release and steroidogenesis. *In vitro* follicle growth is a unique and versatile method for preserving fertility in cancer patients and studying ovarian and follicle biology (Tingen *et al.* 2011, Hornick *et al.* 2013). To date, encapsulation of isolated follicles within alginate hydrogel has been widely used as a 3D follicle culture (Desai *et al.* 2010, Filatov *et al.* 2015, Green & Shikanov 2016). Conventional ovarian tissue culture is a simple method for developing early-stage follicles. However, this method is not applicable for developing late-stage follicles because it cannot maintain normal morphology beyond the preantral stage (Desai *et al.* 2010). To overcome this limitation, we developed a 3D ovarian tissue culture system supported by Matrigel (BD Biosciences, USA) (Higuchi *et al.* 2015). Matrigel is a mouse tumor cell-derived natural hydrogel comprising several extracellular matrix (ECM) proteins and growth factors (Hughes *et al.* 2010). Immature ovarian tissue pieces cultured on Matrigel significantly enhanced antral cavity formation and oocyte competence compared with those cultured without it (Higuchi *et al.* 2015). Furthermore, 3D culture-derived oocytes developed into full-term offspring after *in vitro* maturation (IVM) and *in vitro* fertilization (IVF) (Higuchi *et al.* 2015). This significant advancement in our 3D culture system has the potential to revolutionize the field of *in vitro* follicle culture and its applications.

Cell adhesion is one of the typical biological functions of the ECM to control cell proliferation, migration and differentiation (Nuttelman *et al.* 2001, Zhu 2010). The ECM provides various cell-adhesive domains for binding cell surface receptors to regulate cell functions. Integrins are the major cell surface receptors responsible for cell attachment to the ECM (Haas & Plow 1994). The triple Arg-Gly-Asp (RGD) peptide sequence was identified as a minimal integrin-binding motif in fibronectin (Pierschbacher & Ruoslahti 1984) and in many other ECM proteins (Suzuki *et al.* 1985, Oldberg *et al.* 1986, Grant *et al.* 1989). The RGD motif is known as the most effective and commonly used ECM-derived bioactive molecule for cell-adhesive modification (Hersel *et al.* 2003, Zhu 2010). To examine the effect of RGD on follicle growth, we recently established a 3D ovarian tissue culture supported by dextran hydrogel (Matsushige *et al.* 2022). Bacterial-derived dextran lacks cell-specific adhesion in nature (Sun *et al.* 2010, Sun & Mao 2012). Therefore, non-bioactive dextran hydrogel could provide an ideal condition for evaluating the effect of individual factors (single and multiple) on follicle growth *in vitro*. In our study, the ovarian tissues cultured in RGD-modified dextran hydrogel significantly promoted antral follicle development and estradiol production compared with those cultured in dextran hydrogel alone (Matsushige *et al.* 2022). Furthermore, follicular oocytes grown in the RGD-modified dextran gel, but not in the dextran alone, successfully developed into blastocyst embryos after IVM and IVF (Matsushige *et al.* 2022). Our results strongly suggested that the RGD peptide is a powerful bioactive molecule that stimulates antral follicle development in our 3D culture system.

The RGD peptide binds to integrins as a ligand to activate various cell functions. Integrins are transmembrane receptors for cell adhesion to the ECM and play important roles in cell–cell interaction (Hynes 2002, Barczyk *et al.* 2010). The integrin family is composed of ⍺β heterodimeric associations; eight β subunits can assort with 18 ⍺ subunits to form 24 distinct integrins (Hynes 2002, Barczyk *et al.* 2010). The RGD motif is recognized only by the eight integrins (⍺vβ1, ⍺vβ3, ⍺vβ5, ⍺vβ5, ⍺vβ8, ⍺5β1, ⍺IIBβ3 and ⍺8β1) (Barczyk *et al.* 2010, Ludwig *et al.* 2021). In the ovary, various integrin subunits are expressed in theca cells, interstitial cells, granulosa cells or oocytes in a stage-specific manner (Almeida *et al.* 1995, Nakamura *et al.* 1997, Burns *et al.* 2002, Monniaux *et al.* 2006, Itami *et al.* 2011).

One of the advantages of the 3D ovarian tissue culture is maintaining an intact ovarian environment, including the ovarian interstitial cell population, which occupies the space between the follicles. It has been known that theca cells are differentiated from the interstitial cell population at the early secondary follicle stage to form theca cell layers around the follicles (Young & Mcneilly 2010, Liu *et al.* 2015, Candelaria *et al.* 2019). Theca cells function in diverse roles during folliculogenesis: to synthesize androgens, prepare the crosstalk with granulosa cells and oocytes and provide structural support for the growing follicles (Young & Mcneilly 2010, Richards *et al.* 2018). Theca external fibroblasts form the outermost layers, and theca internal endocrine cells synthesize androgens that act as substrates for estrogen production in granulosa cells and antrum formation (Magoffin & Erickson 1982, Magoffin 2002, Young & Mcneilly 2010, Liu *et al.* 2015, Candelaria *et al.* 2019). Besides their role as substrates for estradiol production, androgens directly regulate follicle development through androgen receptors (ARs). Sen & Hammes (2010) generated a granulosa cell-specific AR knockout model to demonstrate that androgen activities regulate preantral follicle growth and prevent follicular atresia and antral follicle formation (Sen & Hammes 2010). They also found that androgens enhance follicle-stimulating hormone (FSH) receptor expression in granulosa cells, which then augments FSH-mediated follicle development (Sen *et al.* 2014). The effects of theca cells on secondary follicle growth have been reported in isolated follicle culture systems (Cortvrindt *et al.* 1998, Itami *et al.* 2011, Tingen *et al.* 2011, Shiomi-Sugaya *et al.* 2015).

In the present study, we hypothesized that RGD promotes antral follicle development in ovarian tissues through RGD-binding integrins in our 3D culture system. First, we determined the target cell type and target integrins of the RGD peptide in the immature ovary. Then, using the integrin-specific inhibitor, we examined the effects of RGD–integrin interaction on antral follicle development and oocyte competence in the 3D ovarian tissue culture. Finally, we assessed if RGD–integrin interaction plays a role in theca cell differentiation and theca layer formation using the 3D culture of the ovarian interstitial cells.

## Materials and methods

### Mice and treatment

Adult mice were purchased from Charles River (USA). CD-1 females at 2–5 months old were mated with B6D2F1 males at 2–7 months old to obtain (CD-1 × B6D2F1) female pups. After birth, we set one litter as eight newborn pups per mother. Prepubertal ovaries were collected from pups at 14 days postpartum (dpp). We also used a tamoxifen (TAM)-dependent Cre-inducible transgenic mouse model. The following strains of mice were intercrossed: B6.Cg-Gt(ROSA)26Sor^tm14(CAG-tdTomato)Hze^/J termed (R26R^tdT^) (Jackson Labs #007914) (Madisen *et al.* 2010) and *Gli1*^*tm3(cre/ERT2)Alj*^*/J* termed (*Gli*^*CreERT2*^) (Jackson Labs #007913) (Ahn & Joyner 2004). *Gli*^*CreERT2*^ mice were bred to R26R^tdT^ mice. *Gli1*^*CreERT2/+*^*; R26R*^*tdT/tdT*^ males were crossed to CD1 females to generate *Gli1*^*CreERT2*^*; R26R*^*tdT*^ termed (*Gli1/tdT*). *Gli1/tdT* pups were injected intragastrically with 150 μg/g body weight TAM (Chemodex Limited, UK) in sunflower oil one time at 3 dpp, and ovaries were collected at 14 dpp for coculture experiments. All relevant experimental proposals were reviewed and approved by the Institutional Animal Care and Use Committee of the University of Hawaii.

### Media

The basic media used in this study were the same as previously described (Matsushige *et al.* 2022). The ovarian tissues and cells were dissected in Hepes-αMEM (Gibco, USA) supplemented with 5% fetal bovine serum (FBS; Hyclone Laboratories, USA). Ovarian tissues were cultured using an *in vitro* growth (IVG) medium. MEM Alpha GlutaMAX medium (Gibco) was supplemented with 5% FBS, 100 mIU/mL rhFSH (Merck, USA), 5 μg/mL insulin, 5 μg/mL transferrin and 5 ng/mL selenium (Gibco). For ovarian interstitial cell culture, 10% FBS was supplemented in the IVG medium. Cilengitide (Ci) (MedChemExpress, USA) is a high-affinity ⍺vβ3 and ⍺vβ5 integrin antagonist (Mas-Moruno *et al.* 2010, Reardon & Cheresh 2011). Ci was added to the IVG medium at 1, 3 or 10 μM (Ci 1, Ci 3 and Ci 10 respectively). The IVM medium consisted of αMEM supplemented with 5% FBS, 100 mIU/mL rhFSH and 5 ng/mL rhEGF (Promega, USA).

### Preparation of dextran hydrogels

According to the manufacturer’s instructions, we prepared 2.25 mmol/L dextran hydrogels (TRUE7: Sigma-Aldrich, USA) as previously described (Matsushige *et al.* 2022). To synthesize RGD-modified dextran hydrogel, 0.5 mmol/L RGD (TRUERGD, Sigma-Aldrich) was mixed with basic dextran hydrogel (R+). As a control without RGD peptide, 0.5 mmol/L thioglycerol (TRUETHIO, Sigma-Aldrich) was mixed with basic dextran hydrogel (R−). After incubating at room temperature, 5 μL of the gel was placed onto a floating membrane filter (0.4 μm) (HTTP; Millipore Sigma) on IVG medium.

### 3D ovarian tissue culture

In the first experiment, 14 dpp ovaries were cultured under two conditions (cut piece and intact). For the cut piece culture, one ovary was cut into eight pieces and one tissue piece was placed on top of each gel drop (Higuchi *et al.* 2015, Matsushige *et al.* 2022). For the intact culture, one whole ovary was placed on top of the drop. These tissues were cultured under the (R−) or (R+) condition for 7 days at 37 °C, 5% CO_2_ in air. To examine the role of RGD–integrin interaction in follicle growth, the ovarian cut pieces were cultured with or without the Ci inhibitor under five conditions (R−, R+, R + Ci 1, R + Ci 3 and R + Ci 10) for 4 and 7 days. After culturing, the gel drops were treated with dextranase (TRUEENZ, Sigma-Aldrich) to recover the tissues individually for various analyses. Half of the culture media was replaced every other day. We repeated the experiments at least five times per condition.

### 3D culture of ovarian interstitial cell aggregates

The ovarian interstitial cell mass was mechanically isolated from 14 dpp ovaries. The isolated cell mass did not contain follicles, granulosa cells and oocytes. After centrifugation, each cell aggregate (around 1 × 10^4^ cells) was cultured under three conditions (R−, R+ and R + Ci 10) for 7 days. These aggregates were examined using an Olympus BX41 fluorescence microscope (Japan). Half of the culture media was replaced every other day. We repeated the experiments at least five times per condition.

### 3D coculture of ovarian interstitial aggregate and follicles

To examine the theca layer formation, the interstitial cell aggregates were cultured with follicles in the 3D system. Two to three layered secondary follicles (90–110 μm) were mechanically isolated from wild-type 14 dpp ovaries. Healthy follicles that contained minimal theca cells around the basement membrane were selected. The interstitial cell aggregates were prepared from the *Gli/tdT* ovaries at 14 dpp. Two follicles and one aggregate were placed in one hydrogel drop next to each and cocultured under three conditions (R−, R+ and R + Ci 10) for 7 days. The samples were examined using an Olympus BX41 fluorescence microscope (Japan) and a Leica SP8 confocal microscope (Germany). Half of the culture media was replaced every other day. We repeated the experiments at least five times per condition.

### Ovary-specific mRNA expression of integrin subunits

Eight RGD-binding integrins consist of nine integrin subunits (⍺v, ⍺5, ⍺8, ⍺IIB, β1, β3, β5, β6 and β8). Total RNA was extracted from individual ovaries of four different mice at 14 dpp using a Direct-zol RNA Miniprep system (Zymo Research, USA). The cDNA was synthesized using a QuantiTect Reverse Transcription Kit (Qiagen, Germany). The primer sequences are shown in Supplemental Table 1 (see section on Supplementary materials given at the end of the article). After PCR using a PTC-100 thermal cycler (MJ Research, USA), 4 μL of each product were loaded onto 2% agarose gel for electrophoresis.

### Immunohistochemistry

Immunohistochemical localization of integrin subunits (αv, β1, β3 and β5) was examined on 14 dpp and 21 dpp ovaries. Frozen sections were incubated with a primary antibody against αv, β1, β3 or β5 in 1% bovine serum albumin/phosphate-buffered saline (Supplemental Table 2). After washing, the sections were incubated for 1 h with the secondary antibody (Supplemental Table 2). Cell nuclei were stained with 4′,6′-diamidino-2-phenylindole (Thermo Fisher Scientific, Waltham, Massachusetts, USA). These samples were examined using an Axio Scope 2 Plus fluorescence microscope (Carl Zeiss Microimaging, Germany). We repeated the experiments at least three times for each integrin subunit.

### *In vitro* follicle and oocyte growth

During the tissue culture, the diameters of the five (on day 4) or three (on day 7) largest follicles in each tissue piece were measured individually using CellSens imaging software (Olympus, Japan) (Matsushige *et al.* 2022). These follicles were then punctured to obtain germinal vesicle (GV)-stage oocytes. Under an inverted microscope (Olympus), the diameter of each oocyte was measured with an ocular micrometer. Analysis of the follicle and oocyte diameter was repeated at least five times for each condition.

### IVM

GV-stage oocytes isolated on day 7 of culture were subjected to IVM. The oocytes surrounded by granulosa cells were cultured in IVM media for 17–18 h. After IVM, surrounding granulosa cells were mechanically removed to categorize oocytes into four statuses: no resumption of meiosis (GV), incomplete resumption of meiosis (GV breakdown: GVBD), resumption and completion of meiosis to metaphase II with a first polar body (MII) or degenerated oocytes (Deg). IVM was repeated at least five times for each condition.

### Quantitative gene expression analysis

On day 7 of culture, the tissue pieces or the interstitial cell aggregates were subjected to analysis of the mRNA levels of steroidogenic-related genes: *Cyp19a1* (cytochrome P450, family 19, subfamily a, polypeptide 1), *Cyp11a1* (cytochrome P450, family 11, subfamily a, polypeptide 1), *Star* (steroidogenic acute regulatory protein), *Lhcgr* (luteinizing hormone/choriogonadotropin receptor), *Fshr* (follicle stimulating hormone receptor), *Hsd3β1* (3β-hydroxysteroid dehydrogenase isoform 1) and *Actb* (β-actin) (Supplemental Table 1). Freshly isolated 14 dpp ovarian tissues and interstitial cells were also used for the qPCR analysis. Total RNA was extracted from each tissue sample followed by cDNA synthesis using the same methods mentioned above. Each cell aggregate was subjected to cDNA synthesis using a SuperScript III Cells Direct cDNA synthesis kit (Invitrogen, USA). qPCR analysis was performed using a MyiQ Single-Color Real-Time PCR Detection System (Bio-Rad Laboratories, USA). The results were normalized to levels detected for *Actb* gene expression. The analysis was repeated at least four times per culture condition.

### Statistical analysis

Data are represented as mean ± standard error of the mean. A paired *t*-test or one-way analysis of variance followed by Tukey’s multiple comparisons test was used to analyze follicle and oocyte sizes and quantitative gene expression levels. The chi-square or Fisher’s exact test was used to analyze categorical data for IVM.

## Results

### Direct contact of RGD to ovarian interstitial cells is necessary for antral follicle growth

**Figure 1:**
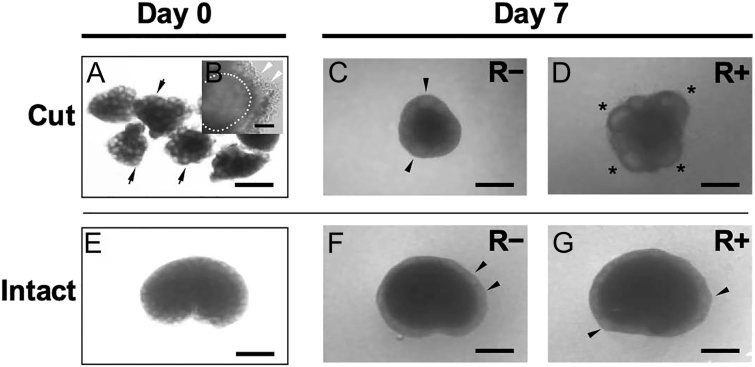
Morphological analysis of follicle growth in cut or intact ovarian tissues in 3-D culture system. The ovarian cut pieces or intact ovaries without cutting were cultured with or without RGD (R+, R−) in the 3-D culture condition. One ovary was cut into 8 pieces which contain multilayered secondary follicles (arrows) (A). After cutting, the ovarian interstitial cell mass became exposed (white arrowheads). One follicle was shown as a dotted circle (B). On day 7, cut pieces cultured in (R−) condition formed preantral follicles (C, arrowheads). In contrast, cut pieces cultured in (R+) condition developed large antrum follicles (D, *). When intact ovaries (E) were cultured without cutting, no antrum formation occurred in (R+) (G, arrowheads) as well as in (R−) (F). Regular bar = 500 μm, short bar = 50 μm.

In our 3D ovarian tissue culture, one ovary was cut into eight pieces (Fig. 1A) and one piece was placed on a hydrogel drop (Higuchi *et al.* 2015, Matsushige *et al.* 2022). By cutting the ovary into small pieces, the ovarian interstitial cell mass between the follicles became exposed (Fig. 1B). On day 7 of culture, cut pieces cultured in RGD-modified dextran hydrogel (R+) significantly promoted antrum formation (Fig. 1D) compared with those in dextran hydrogel alone (R−) (Fig. 1C) (Matsushige *et al.* 2022). Therefore, we hypothesized that the interstitial cell population is the direct target of the RGD peptide to enhance antral follicle development in culture. To test our hypothesis, 14 dpp intact ovaries without cutting (Fig. 1E) were cultured in dextran hydrogels with or without the RGD peptide. Under this condition, hydrogels are in direct contact with the germinal epithelium, an outermost membrane of the ovary. After culturing, antrum formation was rarely observed in the intact ovaries even in the presence of RGD (Fig. 1G), very similar to those cultured without RGD (Fig. 1F). These results suggest that the direct interaction between the RGD peptide and the ovarian interstitial cells is necessary to promote the transition from secondary to antral follicle stage in our 3D culture system.

### RGD-binding integrins expressed in ovarian interstitial cells

**Figure 2:**
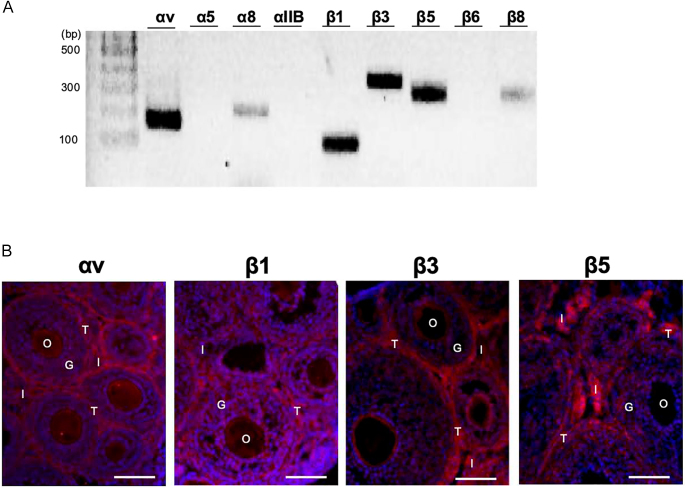
RGD-binding integrin expression in immature ovary. To determine ovary-specific RGD-binding integrins, mRNA levels of 9 integrin subunits in immature ovaries were analyzed in 2% agarose gel. Four integrin subunits (⍺v, β1, β3, and β5) were predominantly expressed (A). Based on this result, we examined immunohistochemical localization of these four subunits in immature ovaries (red) (B). Integrin subunits ⍺v, β3, and β5 were typically localized in the interstitial cell mass and theca cell layers. In contrast, β1 was entirely detected in the ovarian tissues. DAPI counterstaining showed nuclei (blue). G, granulosa cells; I, interstitial cells; O, oocyte; T, theca cells. Bar = 100 μm.

As a ligand, RGD motif binds to eight integrins (Barczyk *et al.* 2010, Ludwig *et al.* 2021). To identify integrins typically expressed in 14 dpp ovaries, we examined mRNA levels of RGD-binding integrin subunits (Fig. 2A). We found that four integrin subunits (⍺v, β1, β3 and β5) were predominantly expressed in 14 dpp ovaries. Next, we determined the localization of these four subunits in the ovaries using immunochemical staining (Fig. 2B). In immature ovaries, ⍺v, β3 and β5 subunits were predominantly localized in the ovarian interstitial area and theca cell layers. In contrast, the β1 subunit was extensively recognized in the entire ovarian tissues, such as oocytes, granulosa cells, interstitial cells and theca cell layers (Fig. 2B). These results demonstrated that integrins ⍺vβ3 and ⍺vβ5 are predominantly localized in the ovarian interstitial compartment. Therefore, we hypothesized that RGD peptide directly interacts with integrins ⍺vβ3 and ⍺vβ5 expressed in the ovarian interstitial cell mass to promote antral follicle development in our 3D ovarian tissue culture.

### Effect of RGD–integrin interaction on follicle growth and cell migration in 3D ovarian tissue culture

**Figure 3:**
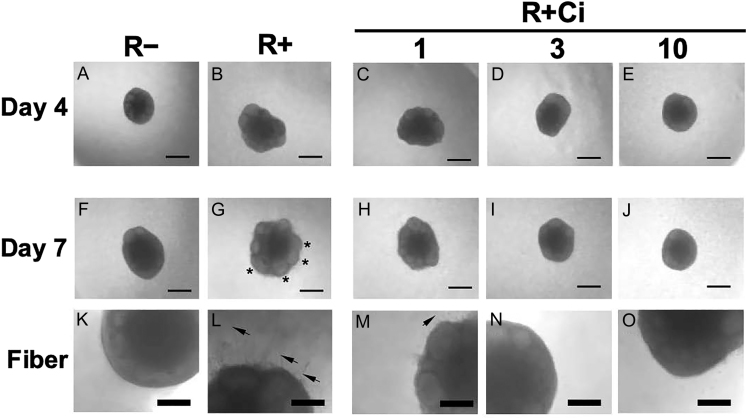
Effect of RGD–integrin interaction on follicle growth in 3-D ovarian tissue culture. Cilenditide (Ci) is a specific inhibitor for integrins ⍺vβ3 and ⍺vβ5. To detect the effects of RGD–integrin interaction on follicle growth, the ovarian tissue pieces were cultured under the five conditions (R−, R+, R + Ci 1, R + Ci 3, and R + Ci 10), respectively. On day 4, the (R+) condition promoted follicle growth (B) compared with the (R−) condition (A). RGD-induced follicle growth was suppressed in the (R + Ci) conditions in a dose-dependent manner (C and E). On day 7, antral follicle growth (G, *), fiber-extension and cell migration (L, arrows) occurred in (R+), but not in (R−) (F and K). In the (R + Ci) conditions, RGD-induced follicle growth (H, I, and J), fiber extension and cell migration (M−O) were gradually suppressed in a dose-dependent manner. Regular bar = 500 μm, bold bar = 200 μm.

**Figure 4:**
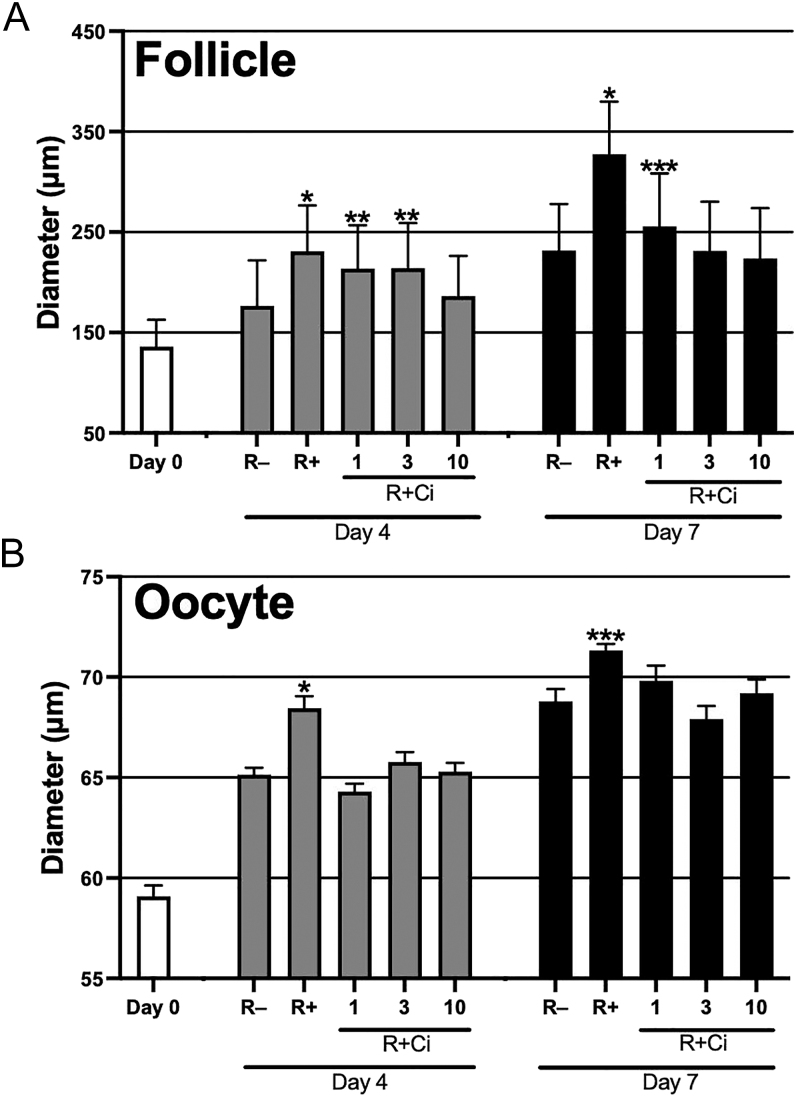
Effect of RGD–integrin interaction on follicle and oocyte growth in 3-D ovarian tissue culture.The ovarian tissue pieces were cultured under the five conditions (R−, R+, R + Ci 1, R + Ci 3, and R + Ci 10). On day 4, the diameters of the five largest follicles per tissue were measured in each condition (*n* = 75–100) (A). On day 7, the diameters of the three largest follicles per tissue were measured (*n* = 66–75) (A). GV-stage oocytes were isolated from these follicles (*n* = 45–80) to measure the oocyte diameters (B). Data represents the mean ± SEM. *P < 0.05 vs. (R−, R + Ci 1, R + Ci 3, and R + Ci 10), **P < 0.05 vs (R− and R + Ci 10), ***P < 0.05 vs (R−, R + Ci 3, and R + Ci 10).

Ci is an integrin inhibitor specific for ⍺vβ3 and ⍺v5 (Mas-Moruno *et al.* 2010, Reardon & Cheresh 2011). To determine the effect of RGD–integrin interaction on antral follicle development, ovarian tissue pieces were cultured in the RGD-modified dextran gel supplemented with Ci at 1, 3 and 10 μM (Fig. 3). When the tissues were cultured with RGD, multiple secondary follicles initiated their growth by day 4 and promoted antrum formation on day 7 of culture (Fig. 3B and G). Another typical function of RGD is stimulating the mobility of the ovarian interstitial cells (Matsushige *et al.* 2022). Under the (R+) condition, fibroblast extension and cell migration were radially observed from the tissues into the surrounding hydrogel matrix (Fig. 3L). Importantly, Ci treatment dramatically suppressed both RGD-induced antral follicle development (Fig. 3C, D, E, H, I and J) and RGD-induced cell migration (Fig. 3M, N and O) in a dose-dependent manner, as to the same level as those under the (R−) condition (Fig. 3A, F and K). Next, we measured the diameters of the large grown follicles in each tissue piece (Fig. 4A). The average follicle diameters of the (R+) condition were significantly larger than those cultured without RGD both on day 4 (231 vs 177 μm) and day 7 (328 vs 232 μm) (both *P* < 0.05). The follicle diameter of (R + Ci) conditions decreased on day 4 of culture. On day 7, (R + Ci) conditions significantly diminished follicle growth in a dose-dependent manner to become similar in size to follicles grown without RGD. Our results strongly suggested that the RGD–integrin ⍺vβ3/⍺v5 interaction is necessary to promote antral follicle development in the 3D ovarian tissue culture.

### Effect of RGD–integrin interaction on oocyte growth and IVM in 3D ovarian tissue culture

We measured the diameters of oocytes isolated from the large grown follicles (Fig. 4B). Under the (R+) condition, the average oocyte diameters were significantly larger than those grown without RGD on day 4 (68.5 vs 65.1 μm) and on day 7 (71.3 vs 68.8 μm) (both *P* < 0.05). In contrast, Ci treatment suppressed RGD-induced oocyte growth regardless of the concentration. On day 7, the diameters of oocytes grown under the (R + Ci 3) and (R + Ci 10) conditions were very similar to those obtained without RGD. To test the oocyte maturity, the GV oocytes isolated from day 7 follicles were subjected to IVM (Table 1). Without RGD, less than half of the GV oocytes were matured at MII. In the presence of RGD, the IVM rate was significantly enhanced up to 78% (*P* < 0.05). As expected, the RGD-induced IVM rate was significantly diminished by the Ci treatment (R + Ci) in a dose-dependent manner (*P* < 0.05). In conclusion, RGD–integrin interaction is necessary to grow follicular oocytes properly in our culture system.

**Table 1:** IVM of oocytes grown in cultured ovarian tissues.

Condition	No. of GV oocytes	No. of oocytes after IVM			
		Deg.	GV	GVBD	M II (%)
R−	63	4	12	18	20 (46.0)^b^
R+	150	1	20	12	117 (78.0)^a^
R + Ci 1	57	3	12	8	34 (59.6)^b^
R + Ci 3	68	3	13	12	40 (56.8)^b^
R + Ci 10	56	6	12	9	29 (51.8)^b^

^a,b^Values with different superscripts within the same column are significantly different (*P* < 0.05).

Ci, cilengitide; GV, germinal vesicle; GVBD, GV breakdown; IVM, *in vitro* maturation; Deg, degenerated oocytes.

### Effect of RGD–integrin interaction on steroidogenesis in 3D ovarian tissue culture

**Figure 5:**
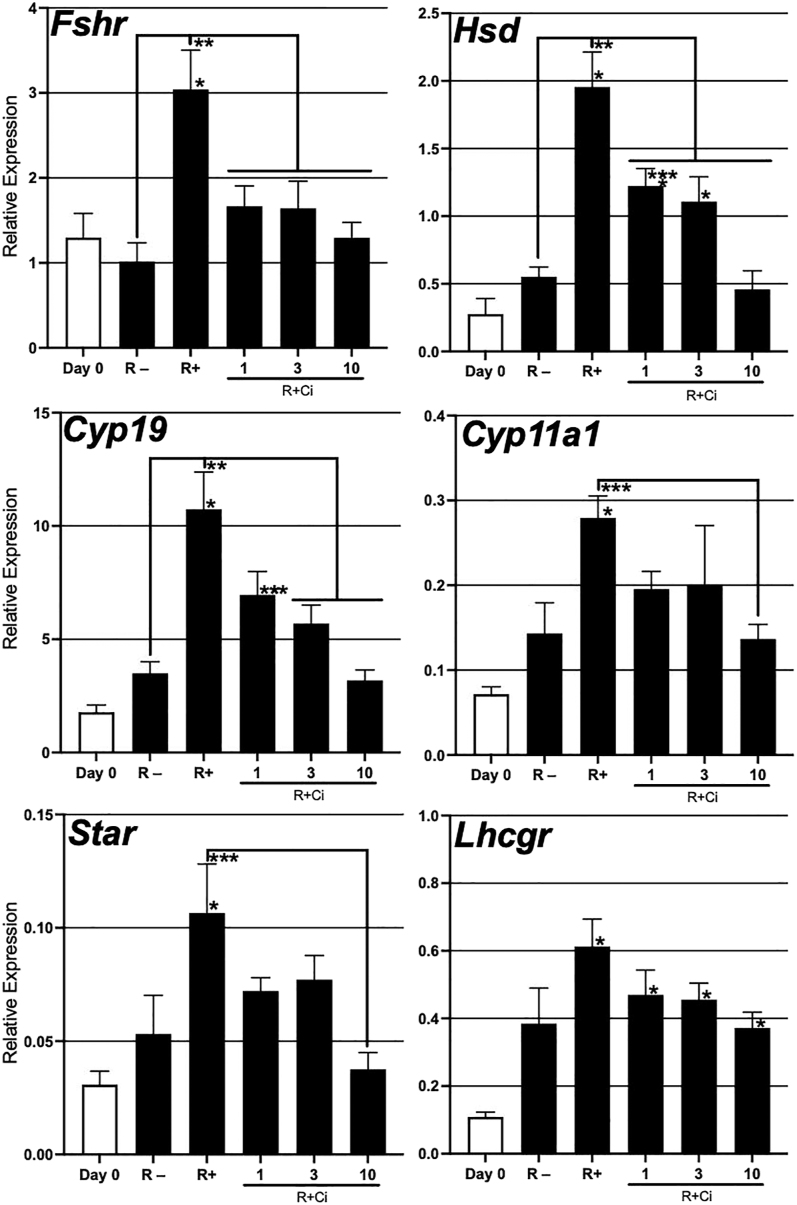
Effect of RGD–integrin interaction on mRNA expression levels of steroidogenic-related genes in 3-D ovarian tissue culture. On day 7 of culture, each ovarian tissue piece cultured under the five conditions (R−, R+, R+ Ci 1, R+ Ci 3, and R+ Ci 10) was subjected to qPCR to determine the mRNA levels of six steroidogenic-related genes. Fresh ovarian tissues at 14 dpp were also analyzed as a day 0 control. The expression levels were normalized to the β-actin mRNA expression. Data represent the mean ± SEM (*n* = 5–18). *P < 0.05 vs day 0, **P < 0.05, ***P < 0.05 vs (R + Ci 10).

To examine whether RGD–integrin interaction regulates sex steroid hormone production, we analyzed the mRNA expression levels of gonadotropin receptors (*Fshr* and *Lhcgr*) and steroidogenic pathway genes (*Cyp11a1*, *Cyp19a1*, *Star* and *Hsd3β1*) in the ovarian tissues (Fig. 5). On day 7 of culture without RGD treatment, the expression levels of six genes were similar to those before culture (day 0) (*P* > 0.05). In contrast, the tissues cultured with RGD significantly upregulated all gene expressions compared with those on day 0 (*P* < 0.05). In addition, these tissues with RGD demonstrated significantly (*Fshr*, *Hsd* and *Cyp19*; *P* < 0.05) or much higher mRNA levels of the genes than those without RGD. These RGD-induced gene activations were entirely inhibited by Ci treatment (R + Ci) in a dose-dependent manner. In particular, RGD-induced mRNA levels of all genes except *Lhgcr* were significantly suppressed under the (R + Ci 10) condition (*P* < 0.05). These results suggested that RGD–integrin interaction promotes the synthetic process of sex steroid hormones in cultured ovarian tissues.

### Effect of RGD–integrin interaction on cell migration and theca cell differentiation in 3D culture of interstitial cell aggregates

**Figure 6:**
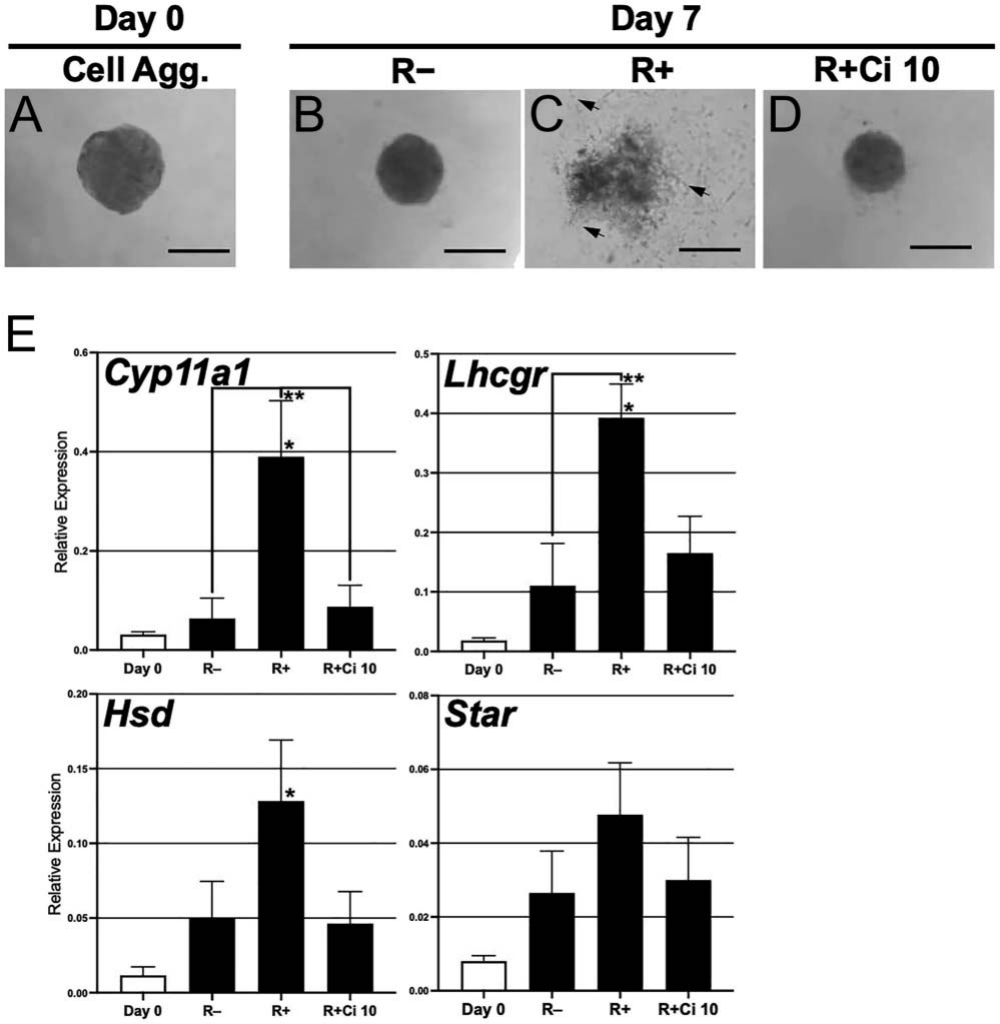
Effect of RGD–integrin interaction on cell migration and theca cell differentiation in 3-D culture of interstitial cell aggregates. The interstitial cell aggregates isolated from immature ovaries (A) were cultured under the three conditions (R−, R+, and R + Ci 10). On day 7 of culture, the aggregates in the (R−) condition formed a compacted and round sphere (B). In the presence of RGD (R+), fiber extension and cell migration were activated (C, arrows). The RGD-induced cell activities were entirely suppressed in the (R+ Ci 10) condition (D). These aggregates were subjected to qPCR analysis to determine the mRNA levels of four steroidogenic-related genes (E). Day 0 cell aggregates were also analyzed as a control. The expression levels were normalized to the β-actin mRNA expression. Data represent the mean ± SEM (*n* = 4–12). *P < 0.05 vs day 0, **P < 0.05.

At the secondary follicle stage, theca cells are differentiated from the theca precursors in the interstitial cell compartment (Young & Mcneilly 2010, Liu *et al.* 2015, Richards *et al.* 2018). To examine the direct effect of RGD–integrin interaction on theca cell differentiation, small aggregates of ovarian interstitial cells were cultured under the three conditions (Fig. 6A). On day 7, the cell aggregates without RGD compacted in size and formed a round sphere (Fig. 6B). In the presence of RGD, the aggregates expanded their area, and fibroblast-like cells and the other types of the cells radially migrated out from the aggregates into the hydrogel matrix (Fig. 6C). Importantly, RGD-induced typical cell migration was completely suppressed by Ci treatment. Under the (R + Ci 10) condition, the cell aggregates formed a round and compacted sphere (Fig. 6D), the same as those without RGD. Next, we analyzed the mRNA expression of four steroidogenic-related genes (*Cyp11a1*, *Lhcgr*, *Hsd* and *Star*), which express in theca endocrine cells (Fig. 6E). On day 7, the RGD-treated aggregates significantly increased mRNA levels of three genes (*Cyp11a1*, *Lhcgr* and *Hsd*) compared with those on day 0 (*P* < 0.05). Under the (R+) condition, all the gene expression levels were significantly (*Cyp11a1* and *Lhcgr*; *P* < 0.05) or dramatically (*Hsd* and *Star*; *P* > 0.05) higher in the aggregates compared with those without RGD (R−). The RGD-induced gene expression was entirely downregulated by Ci treatment to be the same levels without RGD. In conclusion, it was strongly suggested that RGD–integrin interaction directly plays a role in theca cell differentiation in the ovarian interstitial cells *in vitro*.

### Effect of RGD–integrin interaction on theca layer formation in 3D coculture of ovarian interstitial aggregates and follicles

**Figure 7:**
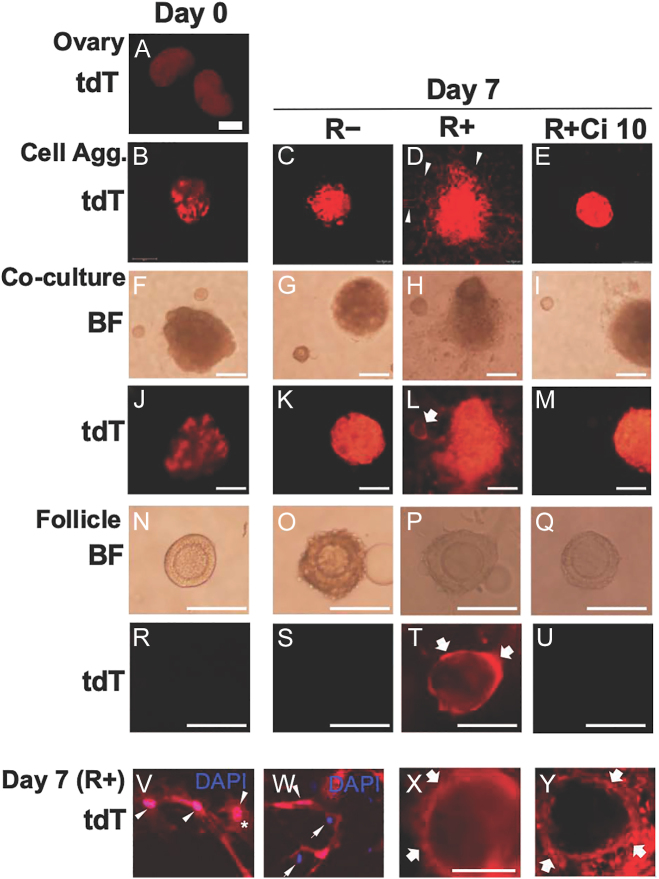
Effect of RGD–integrin interaction on theca layer formation in 3-D co-culture of interstitial cell aggregates and follicles. After TAM injection, ovaries were isolated from *Gli1*/*tdT* mice at 14 dpp (A) to make interstitial cell aggregates (B). On day 7 of culture, the aggregates in (R−) were compacted (C). In the (R+) condition, *tdT*-expressing cells were radially migrated into the hydrogel (D, arrowheads). In contrast, RGD-induced cell migration was completely suppressed in the (R+ Ci 10) condition (E). One *Gli*/*tdT*-derived aggregate and two wild-type secondary follicles (F, J, N, R) were co-cultured for 7 days under the three conditions. In the (R+) condition (H, L, P, T, X, Y), some *tdT*-positive migrating cells surrounded the follicles to form the outermost cell layers, which closely resembled theca layers (L, T, X, Y, thick arrows). In contrast, theca layer formation did not occur in the (R−) (G, K, O, S) or (R+ Ci 10) (I, M, Q, U). On Day 7 in the (R+) condition, *tdT*-positive migrating cells consisted of different cell types such as fibroblast-like cells and round cells (*) with *tdT*-positive (arrowheads) or negative nuclei (arrows) (V, W). Bold bar = 500 μm. Short bar = 200 μm. Regular bar = 100 μm. Confocal images at ×100 (B, C, D, E), ×200 (Y) and ×400 (V, W).

We examined if RGD–integrin interaction plays a role in theca layer formation in the 3D system. For this purpose, the interstitial aggregates prepared from *Glil/tdT* ovaries were cocultured with wild-type early-stage secondary follicles under the three conditions (Fig. 7). It has been well recognized that the cells expressing *Gli1* contribute extensively to the theca layer of the follicle, and steroidogenic theca cells are *Gli1*-positive (Wijgerde *et al.* 2005, Russell *et al.* 2007, Ren *et al.* 2009, Spicer *et al.* 2009, Liu *et al.* 2015). *Glil/tdT* ovaries showed a *tdT*-positive area between the follicles, and *tdT*-positive cells were extensively localized within the day 0 interstitial aggregates (Fig. 7A and B). On day 7 in the coculture without RGD, cell migration did not occur in the aggregate, resulting in the cocultured follicles being left alone without any direct contact with the *tdT*-positive cells (Fig. 7C, G, K, O and S). Under the (R+) condition, on the contrary, *tdT*-positive cells were radially migrated out from the aggregates into the surrounding hydrogel matrix (Fig. 7D, H and L). These *tdT*-positive migrating cells varied morphologically and included fibroblast-like and round-shaped cells (Fig. 7V and W). Importantly, some *tdT*-positive migrating cells reached the cocultured follicles and surrounded them to establish the outermost multiple cell layers, which closely resembled theca cell layers (Fig. 7L, P, T, X and Y). The early-stage secondary follicles increased in size when surrounded by the *tdT*-positive cell layers (Fig. 7T, X and Y), suggesting that the theca layer formation promoted follicle growth. When treated with Ci, RGD-induced cell migration was critically blocked; therefore, *tdT*-positive theca layer formation did not occur around the cocultured follicles (Fig. 7E, I, M, Q and U). In conclusion, these results strongly suggest that RGD–integrin interaction stimulates theca cell differentiation and theca layer formation in the interstitial cell population under the 3D culture system.

## Discussion

In this study, we explored the effects of RGD–integrin interaction on folliculogenesis using the 3D culture system. First, we detected that integrins ⍺vβ3 and ⍺vβ5 localized in the ovarian interstitial cells are the RGD target receptors. By blocking RGD–integrin interaction, RGD-induced antrum formation and oocyte competence were significantly suppressed in cultured ovarian tissues. In the 3D culture of interstitial cell aggregates, RGD–integrin interaction promoted theca cell differentiation and theca layer formation. In conclusion, our results suggest that the RGD peptide promotes theca cell differentiation and theca layer formation through integrins ⍺vβ3/⍺vβ5 to enhance antral follicle development in the 3D culture system.

We found that antral follicle growth occurred only when the RGD-modified hydrogel directly contacted the ovarian interstitial compartment in our 3D ovarian tissue culture. Previously it was reported that the isolated follicles encapsulated with RGD-modified gels enhanced follicle growth and/or oocyte maturation compared with those cultured without RGD (Kreeger *et al.* 2006, Zhao *et al.* 2023). However, the mechanism of RGD-induced antral follicle development remained unknown. We hypothesized that the RGD peptide promotes antral follicle development through the RGD-binding integrins typically localized in the ovarian interstitial compartment. In the mammalian ovary, various integrins are expressed in stage- and species-specific manners (Almeida *et al.* 1995, Nakamura *et al.* 1997, Burns *et al.* 2002, Monniaux *et al.* 2006, Itami *et al.* 2011). This study determined that the RGD-binding integrins ⍺vβ3 and ⍺vβ5 are predominantly located in the interstitial cells and theca cell layers in the immature mouse ovaries. To test the effects of RGD–integrin interaction on follicle growth, the ovarian tissues were cultured with a selective inhibitor of ⍺vβ3 and ⍺vβ5, Ci. As expected, Ci treatment blocked RGD-induced antral follicle growth, steroidogenesis and oocyte development, suggesting that the RGD peptide promotes antral follicle development through binding to integrins ⍺vβ3/⍺vβ5 expressed in the interstitial compartment in our 3D culture.

In this study, RGD–integrin interaction typically activated cell migration in the ovarian tissues and interstitial cell aggregates. In response to RGD, fibroblast-like and round-shaped cells were three-dimensionally scattered and migrated into the surrounding hydrogel matrix. Cell migration is a typical function of integrins ⍺vβ3 and ⍺vβ5 through signal transduction pathways (Huttenlocher & Horwitz 2011, Russo *et al.* 2013, Bianconi *et al.* 2016). Our question was if the RGD-activated migrating cells contribute to theca layer formation in the 3D culture. As the follicle develops, the theca cell layer arises from the surrounding interstitial cell mass (Wijgerde *et al.* 2005, Russell *et al.* 2007, Ren *et al.* 2009) by poorly defined mechanisms. Liu *et al.* (2015) determined that theca cells are derived from *Wt1*^*+*^ cells indigenous to the ovary and *Gli1*^*+*^ mesenchymal cells that migrate from the mesonephros (Liu *et al.* 2015). These theca cell precursors acquire the theca lineage marker *Gli1* in response to paracrine signals of the hedgehog ligands secreted from granulosa cells (Liu *et al.* 2015). The cells expressing *Gli1* contribute extensively to the theca layer of the follicle, and steroidogenic theca cells are *Gli1*-positive (Wijgerde *et al.* 2005, Russell *et al.* 2007, Ren *et al.* 2009, Spicer *et al.* 2009, Liu *et al.* 2015). In this study, the interstitial cell aggregates isolated from the *Gli/tdT* ovaries were cocultured with wild-type early-stage secondary follicles. Notably, some of *Gli1*-positive cells attached the basement membrane of cocultured secondary follicles and surrounded them to build the outermost multiple cell layers, like theca cell layers. In this study, the characterization of the *Gli1*-positive cells that contributed to the theca layer formation remained uncertain. Using the *Gli1/tdT* mice, Cowan & Quirk (2021) determined that *Gli1*-expressing precursors in the ovarian mesenchyme contribute to three different cell types (pericytes, vascular smooth muscle cells and steroidogenic theca cells) within the theca layers (Cowan & Quirk 2021). They found that a large population of theca cells consists of vascular-related cell types and around half of them were *Gli1*-positive. In contrast, steroidogenic theca cells occupied only 7% of the total theca cell population, but half of them were *Gli1*-positive in the Tg ovaries (Cowan & Quirk 2021). These data suggest that multiple theca cell precursors are recruited at different times and from different populations to establish the entire theca cell layers.

The theca layers of growing follicles provide a structural framework that is largely composed of ECM factors (Yasuda *et al.* 2005, Richards *et al.* 2018). The migrated cells in response to RGD may bind to the follicular basement membrane through integrin targets to form theca layers in the coculture. Using the 3D coculture system, Itami *et al.* (2011) examined the effect of THY1–integrin β3 interaction on theca layer formation (Itami *et al.* 2011). The glycoprotein THY1 sequence contains the integrin-binding RLD motif, which selectively binds to integrin β3. Both proteins have colocalized in theca cell layers of preantral follicles. The theca internal cells cocultured with preantral follicles in a collagen gel matrix formed multiple theca layers around the follicles (Itami *et al.* 2011). However, anti-THY1 antibody treatment completely suppressed theca layer formation in the coculture (Itami *et al.* 2011). Their results supported our conclusion that the ECM-derived RGD motif interacts with target integrins localized in the ovarian interstitial cells to promote theca cell differentiation and layer formation under the 3D culture system.

In this study, we determined that the ovarian interstitial cells stimulated by the RGD–integrin ⍺vβ3/⍺vβ5 interaction play an important role in antral follicle development and theca cell differentiation. Our 3D culture system could be a useful model to define the roles of individual molecules in folliculogenesis and theca cell differentiation.

## Supplementary material



## Declaration of interest

The authors declare that there is no conflict of interest that could be perceived as prejudicing the impartiality of the work reported.

## Funding

This work was supported by the National Institutes of Health (NIH) Centers of Biomedical Research Excellence (P30GM131944) to YY, the George F. Straub Trust of Hawaii Community Foundation (MedRes_2023_00002690) to YY, the NIH Small Business Innovation Research Grant (R43HD114508-01) to YY and the Kosasa Graduate Student Assistantship Award to CM.

## Author contribution statement

CM contributed to data curation, formal analysis, writing of the original draft, review and editing. KK, AB and MM helped in data curation and formal analysis. MDT contributed to data curation, writing of the original draft, review and editing. YY helped in data curation, formal analysis, writing of the original and revised drafts, review and editing.
