# Supplementary material for: RGD peptide promotes follicle growth through integrins αvβ3/αvβ5 in three-dimensional culture
Source: Reproduction. 2025 Jan 2;169(1):e240151. doi: 10.1530/REP-24-0151 (PMC11729052; doi:10.1530/REP-24-0151)
Supplement: Supplementary file 1 [file supplementary_materials.pdf]

Supplemental Table 1. Primers used for qPCR.

Gene		Sequence (5' to 3')
Integrin αv	F	GGATTCGCCGTGGACTTCTT
	R	CAAACTCAATGGGCTGGCAC
Integrin α5	F	AGGGAAGAGCGGGCACTA
	R	TTGGATGGAACTCAGGCAAC
Integrin α8	F	GGACAGGTCTACTTGTACCTTCAAG
	R	CATTGTAAATGAGGACTTTACCTCG
Integrin αIIβ	F	GAGGTGGGCCGCGTTTATTT
	R	ACAGCACCACGAAGGGAGAA
Integrin β1	F	ATGCCAAATCTTGCGGAGAA
	R	CATCGTGCAGAAGTAGGCATT
Integrin β3	F	TGCTTACGGGAAAATCCGCT
	R	CCCACACTCAAAAGTCCCGT
Integrin β5	F	TCTTCTTCACTGCCACCTGC
	R	AGCCCACAGGTGTATGTTCC
Integrin β6	F	GTCTGGCTCCCGGCTGGC
	R	AGTTAATGGCAAAATGTGCT
Integrin β8	F	CTGAAGAAATACCCCGTGGA
	R	ATGGGGAGGCATACAGTCT
Cyp19a1	F	ATGTCGGTCACTCTGTACTTC
	R	TTTATGTCTCTGTCACCCACAAC
Cyp11a1	F	TCCTTTGAGTCCATCAGCAG
	R	GTCCTTCCAGGTCTTAGTTCT
Star	F	CCACCTGCATGGTGCTTCA
	R	TTGGCGAACTCTATCTGGGTCTG
Lhcgr	F	AAGCACAGTTAGAGAAGCGA
	R	GGTCAGGAGAACAAAGAGGA
Fshr	F	ACGCCATTGAACTGAGATTTG
	R	GAACACATCTGCCTCTATTACC
Hsd3β1	F	CAAGTGTGCCAGCCTTCATCT
	R	GGCCATCAGGACGATCTTAT
B-actin	F	CCTGTATGCCTCTGGTCGTA
	R	CCATCTCCTGCTCGAAGTCT

Supplemental Table 2. Anti-integrin antibodies for immunohistochemistry.

Antibody	Integrin	Manufacturer	Cat. number	Dilution
Primary	αv	R & D Systems	AF1219	1:500
	β1	Proteintech	12594-1-AP	1:500
	β3	Abcam	Ab203122	1:500
	β5	Cell Signaling	3629	1:1500
Secondary	Alexa Fluor 568	Invitrogen	A11057	1:500
	Alexa Fluor 594	Jackson ImmunoResearch Labs	111-585-144	1:500
